# Native T1-mapping detects the location, extent and patterns of acute myocarditis without the need for gadolinium contrast agents

**DOI:** 10.1186/1532-429X-16-36

**Published:** 2014-05-23

**Authors:** Vanessa M Ferreira, Stefan K Piechnik, Erica Dall’Armellina, Theodoros D Karamitsos, Jane M Francis, Ntobeko Ntusi, Cameron Holloway, Robin P Choudhury, Attila Kardos, Matthew D Robson, Matthias G Friedrich, Stefan Neubauer

**Affiliations:** 1 Division of Cardiovascular Medicine, Radcliffe Department of Medicine, University of Oxford, John Radcliffe Hospital, Oxford OX3 9DU, UK; 2 Department of Cardiology, Milton Keynes NHS Hospital Foundation Trust, Milton Keynes MK6 5LD, UK; 3 Montreal Heart Institute, Departments of Medicine and Radiology, Université de Montréal, Montréal, QC H1T 1C8, Canada; 4 Stephenson Cardiovascular MR Centre, Libin Cardiovascular Institute of Alberta, Calgary, AB T2N 2 T9, Canada

**Keywords:** Native T1-mapping, ShMOLLI, Myocarditis, T2-weighted MRI, Cardiovascular magnetic resonance, Late gadolinium enhancement

## Abstract

**Background:**

Acute myocarditis can be diagnosed on cardiovascular magnetic resonance (CMR) using multiple techniques, including late gadolinium enhancement (LGE) imaging, which requires contrast administration. Native T1-mapping is significantly more sensitive than LGE and conventional T2-weighted (T2W) imaging in detecting myocarditis. The aims of this study were to demonstrate how to display the non-ischemic patterns of injury and to quantify myocardial involvement in acute myocarditis without the need for contrast agents, using topographic T1-maps and incremental T1 thresholds.

**Methods:**

We studied 60 patients with suspected acute myocarditis (median 3 days from presentation) and 50 controls using CMR (1.5 T), including: (1) dark-blood T2W imaging; >(2) native T1-mapping (ShMOLLI); (3) LGE. Analysis included: (1) global myocardial T2 signal intensity (SI) ratio compared to skeletal muscle; (2) myocardial T1 times; (3) areas of injury by T2W, T1-mapping and LGE.

**Results:**

Compared to controls, patients had more edema (global myocardial T2 SI ratio 1.71 ± 0.27 vs.1.56 ± 0.15), higher mean myocardial T1 (1011 ± 64 ms vs. 946 ± 23 ms) and more areas of injury as detected by T2W (median 5% vs. 0%), T1 (median 32% vs. 0.7%) and LGE (median 11% vs. 0%); all p < 0.001. A threshold of T1 > 990 ms (sensitivity 90%, specificity 88%) detected significantly larger areas of involvement than T2W and LGE imaging in patients, and additional areas of injury when T2W and LGE were negative. T1-mapping significantly improved the diagnostic confidence in an additional 30% of cases when at least one of the conventional methods (T2W, LGE) failed to identify any areas of abnormality. Using incremental thresholds, T1-mapping can display the non-ischemic patterns of injury typical of myocarditis.

**Conclusion:**

Native T1-mapping can display the typical non-ischemic patterns in acute myocarditis, similar to LGE imaging but without the need for contrast agents. In addition, T1-mapping offers significant incremental diagnostic value, detecting additional areas of myocardial involvement beyond T2W and LGE imaging and identified extra cases when these conventional methods failed to identify abnormalities. In the future, it may be possible to perform gadolinium-free CMR using cine and T1-mapping for tissue characterization and may be particularly useful for patients in whom gadolinium contrast is contraindicated.

## Background

Acute myocarditis can be detected using cardiovascular magnetic resonance (CMR) using multiple tissue characterization techniques, especially T2-weighted (T2W) imaging for edema and late gadolinium enhancement (LGE) [1-3]. LGE is especially powerful in generating excellent contrast between diseased and normal myocardium, allowing visualization of LGE patterns and distribution to differentiate between ischemic and non-ischemic etiologies of myocardial disease [4,5].

However, there are some limitations in using LGE alone in the diagnosis of acute myocarditis. Very small areas of myocarditis may not always be detected. Some patients demonstrate predominant global edema rather than frank myocyte necrosis, resulting in negative findings on LGE imaging [1]. Diffuse myocarditis may not be objectively detectable given that the image analysis of LGE requires normal myocardium as a reference region of interest (ROI) for comparison. Additionally, although the risk is low, gadolinium-based contrast agents are associated with the rare but serious complication of nephrogenic systemic fibrosis in patients with significant renal impairment [6]. Thus, it would be useful if there were a method which is sensitive to displaying the different changes in myocarditis without the need for exogenous contrast agents.

Native (or pre-contrast) T1-mapping directly estimates the T1 (proton spin–lattice) relaxation time of tissues on a pixel-by-pixel basis. Native myocardial T1 values have recently been validated in a large, multicentre normal cohort study to have a tight normal range [7], and shown to be superior to T2-weighted imaging in detecting edema [8] as well as acute myocarditis [9]. Further, native T1-mapping has an equivalent diagnostic performance to LGE in diagnosing myocarditis, with a superior sensitivity (90%) to both T2W and LGE imaging [9]. In addition to providing a single numeric average myocardial T1 value for a subject, T1-maps should be fully utilized for visual and spatial characterization of changes in the left ventricular myocardium.

In this study, the hypothesis is two-fold: (1) given that T1-mapping is highly sensitive compared to conventional CMR techniques in detecting changes in myocarditis [9], we propose that it should be able to directly locate the areas of myocardial involvement on a pixel-wise basis, including additional areas of injury; (2) T1-mapping may be able to detect the non-ischemic patterns of injury typically seen on LGE images as areas with higher T1 values compared to the rest of the myocardium. We demonstrate novel approaches for visualizing the extent and patterns of myocardial injury in acute myocarditis using topographic T1-maps and incremental T1 thresholds.

## Methods

### Characteristics of the study population

This was a prospective study including 60 consecutive patients (age 41 ± 16 years; 25% female) presenting with suspected acute myocarditis [1] as inpatients to two hospitals (The John Radcliffe Hospital and Milton Keynes Hospital, United Kingdom) [9]. Patients underwent CMR scanning at the John Radcliffe Hospital within fourteen days (median 3 days, interquartile range IQR 1–6 days) of presentation between January 2010 and March 2013. In a previously published study, we demonstrated the superior sensitivity of native T1-mapping to T2W and LGE imaging in detecting acute myocarditis [9]. The cumulative CMR data derived from this study and new patient material were utilized for the present analysis to generate topographic and incremental threshold T1-maps. All patients had: (a) acute chest pain; (b) elevation in cardiac troponin I level > 0.04 μg/L (median 4.50 μg/L, IQR 0.89-14.98 μg/L); (c) history of recent systemic viral or inflammatory illness (93%, n = 56/60), or absence of significant (>50%) obstructive coronary artery disease (CAD) on coronary angiography (70%, n = 42/60); coronary angiography was not performed in the remaining patients (n = 18) due to young age <35 years (n = 14), no known risk factors for CAD (n = 2) or a convincing history of a viral/inflammatory etiology (n = 2). Exclusion criteria included contraindications to CMR, previous myocardial infarction, previous myocarditis or any chronic cardiac conditions. Patients who demonstrated myocardial infarction as evidenced by an ischemic pattern of LGE (i.e. an isolated area involving the subendocardium) or an obvious alternative diagnosis on CMR (such as Takotsubo or hypertrophic cardiomyopathy) were also excluded. Healthy volunteers (n = 50) of similar age and gender distribution (age 41 ± 13 years; 26% female) with no cardiac history or known cardiac risk factors, not on cardiovascular medications and with a normal electrocardiogram underwent CMR as controls. Ethical approval was granted for all study procedures and all subjects gave written informed consent.

### Cardiovascular magnetic resonance

CMR studies were performed on a single 1.5 Tesla MR system (Avanto, Siemens Healthcare, Germany) using a 32-channel phased-array coil, except for short-tau inversion recovery (STIR) imaging for which the body coil was used, as previously published [9]. Briefly, cine images were obtained in three long-axis views, and in the short-axis plane covering the base to apex of the heart. Tissue characterization covering the left ventricle from base to apex was performed for T2W, T1-mapping and LGE imaging in matching short-axis slices. Dark-blood T2-weighted imaging for edema was performed using the STIR sequence [1,10] and T1-mapping using the Shortened Modified Look-Locker Inversion recovery (ShMOLLI) sequence [11], before administration of contrast agents. LGE imaging was acquired in the long and short-axis planes using a T1-weighted phase-sensitive inversion recovery (PSIR) sequence [12] 8–12 minutes after intravenous administration of contrast agent (Gadodiamide, Omniscan, GE Healthcare, total 0.13 mmol/kg). Typical imaging parameters were as previously published [8,9] (see Additional file 1).

### Image analysis

Image analysis of left ventricular ejection fraction was performed using Argus software (Siemens Medical Solutions) on cine images. Analysis of short axis images from T1-mapping, T2W and LGE imaging was performed as previously published [8,9]. Briefly, on dark-blood T2W images, edema is diagnosed when myocardial T2 SI ratio is ≥2:1 compared to that of skeletal muscle or when myocardial T2 SI is ≥2.0 standard deviations above the mean SI of remote tissue in the same slice [1]. Remote myocardium in acute myocarditis may be challenging to identify as the process may be global; thus a region of the myocardium with no obvious visual increase in relative T2 SI and no LGE was chosen to represent myocardium least affected by the disease process, taking care to also avoid regions with abnormally low signal. Results of T1-mapping were based on quantitative analysis of all T1-maps (rather than visualization of color maps). Care was taken when placing the endo- and epicardial contours to avoid contamination by blood-pool and extra-myocardial structures to minimize the partial volume effect on myocardial T1 values [7]. Acute myocardial injury is diagnosed when T1 is > 990 ms, as previously published for the objective detection of acute myocardial edema [8]. Focal areas of LGE were defined as those with a SI ≥2.0 standard deviations above the mean SI of remote myocardium. In addition to using T1 > 990 ms to generate topographic maps to delineate the location and extent of myocardial involvement, we also used a set of higher, incremental T1 thresholds to detect and describe the visual pattern of lesions based on their T1 elevation. For all analyses, only myocardial regions with a contiguous area of ≥ 40 mm^2^ above the specified thresholds were considered abnormal [9]; this corresponds to 10 adjacent pixels for the STIR method, in accordance with currently proposed recommendations [1] to reduce the detection of noise as positive findings. To calculate the extent of myocardial injury in a subject detected by the tissue characterization techniques, the percentage of abnormal myocardium as defined above was determined for each segment and then averaged for that subject. In addition to quantitative image analyses, qualitative visual analysis for T2W and LGE images were performed by at least 2 expert CMR cardiologists; any difference in opinion was resolved by presenting the case to at least one additional expert CMR cardiologist before reaching a final interpretation.

### CMR image quality assessment

Each LV myocardial segment was strictly assessed for image quality before inclusion into the final analyses and only segments with no or minimal artifacts were included, as previously published [8]. On cine imaging, 3% of segments corresponding to the LVOT were rejected; on dark-blood T2W imaging, 9% were rejected due to artifacts, signal dropout or breathing motion; T1-maps were assessed for quality in three ways: examination of the T1-map, the raw T1 images and R^2^ maps; 11% of the segments were excluded due to off-resonance artifacts, partial volume effect, poor T1 fit on the R^2^ maps, patient movement or low SNR; on LGE imaging, 3% were rejected due to artifacts caused by patient movement or poor image quality. To avoid bias towards or against any single technique, main analyses were repeated with all artifacts re-included and results provided in the main text or Additional file 1.

### Statistical analysis

Normality of data was tested using the Kolmogorov-Smirnov test. Normally distributed data are presented as mean ± standard deviation (SD); non-parametric data are presented as median with interquartile range (IQR). Unpaired samples between groups were assessed by the unpaired 2-tailed Student’s *t*-test, or the Mann–Whitney *U* test for non-parametric data. Any segmental analysis was averaged on a per-subject basis before any inter-individual and group comparisons to control for clustering of segments within each subject. All statistical tests were two-tailed, with p-values of less than 0.05 considered statistically significant. To determine the presence of significant differences in subject groups when using multiple CMR methodologies, ANOVA analysis was performed with Bonferroni corrected post-hoc comparisons for parametric data; for non-parametric data, the Kruskal-Wallis one-way analysis of variance was performed with post-hoc pairwise comparisons. For comparisons of the extent of myocardial injury measured by multiple CMR methodologies within the same patient subgroup, the Friedman test was used for non-parametric data with post-hoc comparisons. Receiver operator characteristic (ROC) analysis was performed to compare the diagnostic performance of the CMR methods in detecting myocardial changes in patients compared to controls. Significance of ROC analyses was assessed using the method of Delong et al. [13]. The McNemar test and Cochran’s Q-test were used for comparing combinations of CMR tissue criteria in the diagnosis of acute myocarditis. Statistical analyses were performed using MedCalc (version11.5.1.0, MedCalc Software, Mariakerke, Belgium).

## Results

### CMR findings in acute myocarditis

**Figure 1:**
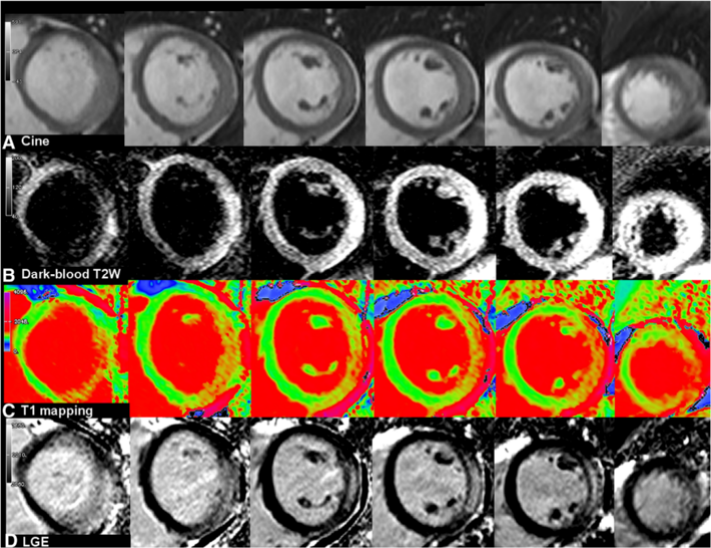
**Whole-heart multiparametric cardiovascular magnetic resonance (CMR) tissue characterization in acute myocarditis.** (Left to right) Short-axis slices covering the left ventricle from base to apex.

An example of whole-heart multiparametric tissue characterization is shown in Figure 1. CMR findings are presented in Table 1. Compared to controls, patients had a significantly lower mean LV ejection fraction (EF), higher global myocardial T2 SI ratio, mean myocardial T1 values and larger areas of myocardial injury measured by T2, T1 and LGE. The pattern of LGE, when each is considered in isolation, was predominantly subepicardial (96%) and midwall (84%), localized most frequently to the lateral wall (98%) and inferior wall (96%), typical for myocarditis. None of the 60 patients demonstrated an isolated ischemic (subendocardial) pattern of LGE to suggest myocardial infarction as the etiology of the acute presentation.

**Table 1:** Tissue characteristics in controls and patient groups

**CMR findings**	**Controls (n = 50)**	**All patients (n = 60)**	**Group I (Edema+, LGE+) (n = 41)**	**Group II (Edema-, LGE+) (n = 12)**	**Group III (Edema-, LGE-) (n = 7)**
Ejection fraction (%)	72 ± 6	64 ± 12*	61 ± 13^†^	70 ± 11	72 ± 6
Global myocardial T2 SI ratio^‡^	1.56 ± 0.15	1.71 ± 0.27*	1.79 ± 0.27^†^	1.60 ± 0.16	1.47 ± 0.17
Myocardial injury by T2 SI ratio ≥ 2.0 (%)	0 (0 to 2)	5 (1 to 18)*	9 (3 to 22)^†^	0.6 (0 to 4)	0 (0 to 0)
Mean myocardial T1 (ms)	946 ± 23	1011 ± 64*	1030 ± 62^†^	986 ± 54^†^	947 ± 27
Myocardial injury by T1 ≥ 990 ms (%)	0.7 (0 to 3)	32 (15 to 65)*	49 (24 to 70)^†^	15 (12 to 29)^†^	6 (2 to 7)^†^
Myocardial injury by LGE (%)^§^	0 (0 to 1) (n = 35)	11 (4 to 20)*	16 (8 to 24)^†^	7 (4 to 12)^†^	1 (0 to 2)

Values are presented as mean ± SD or median (interquartile range). *p < 0.001 when compared to controls.

In post-hoc subgroup analyses: ^†^significantly different from controls;

areas of injury by T2 (edema) were significantly larger in Group I than Group II and III;

areas of injury by both T1 and LGE were significantly larger in Group I > II > III patients.

^‡^Global myocardial T2 SI ratio = T2 SI _myocardium: sk. muscle_

^§^LGE is detected when myocardial SI is ≥ 2.0 standard deviation above the mean SI of remote myocardium.

Receiver operator characteristics (ROC) analysis comparing diagnostic performance of the techniques showed that T1-mapping and LGE (with area-under-the-curve AUC of 0.94 and 0.93, respectively; p = ns) were significantly superior to dark-blood T2W imaging in the detection of acute myocarditis (AUC 0.76; p < 0.05 for both). Repeat analysis including all image artifacts showed preservation of all relative relationships and statistical significance (AUC of 0.92, 0.94 and 0.75, respectively). These results are closely comparable to our previous results using the same techniques in patients with suspected acute myocarditis [9]. The diagnostic performance of all individual and combination tissue criteria is presented in Table 2.

**Table 2:** Diagnostic performance of CMR tissue characterization methods in the detection of suspected acute myocarditis

**Tissue criteria**	**Sensitivity (%)**	**Specificity (%)**	**Accuracy (%)**	**PPV (%)**	**NPV (%)**
**Individual**					
T1-mapping*	90	88	89	90	88
Dark-blood T2*	48	86	66	81	58
LGE	72	97	81	98	67
**Combination (with LGE)**					
Dark-blood T2 and LGE (2 out of 2)^†‡^	45	97	64	96	51
Dark-blood T2 or LGE (Any 1 of 2)	75	86	79	90	67
T1-mapping and LGE (2 out of 2)^†^	67	97	78	98	63
T1-mapping or LGE (Any 1 of 2)	95	83	91	91	91
T1-mapping, dark-blood T2 or LGE (Any 1 of 3)	95	71	86	85	89
T1-mapping, dark-blood T2 or LGE (Any 2 of 3)	70	97	80	98	65
T1-mapping and dark-blood T2 and LGE (3 out of 3)	45	97	64	96	51
**Combination (without LGE)**					
T1-mapping and dark-blood T2 (2 out of 2)^‡^	48	98	71	97	61
T1-mapping or dark-blood T2 (Any 1 of 2)	90	76	84	82	86

*statistically different (p < 0.05); ^†‡^no statistical difference (p = ns). T1-mapping: myocardial injury is detected when T1 is ≥ 990 ms; Dark-blood T2-weighted imaging: edema is diagnosed when the T2 SI ratio (T2 SI _myocardium : skeletal muscle_) is ≥ 2:1; Late gadolinium enhancement (LGE) is detected when myocardial SI is ≥ 2 SD above mean SI of remote myocardium. For each technique, only contiguous areas of myocardium ≥40 mm^2^ above the stated threshold were considered relevant; involvement of ≥5% of any segment on a per-subject basis was the threshold used for comparison of methods. PPV = positive predictive value; NPV = negative predictive value.

### Incremental value of T1-mapping in the detection of myocardial injury in acute myocarditis

**Figure 2:**
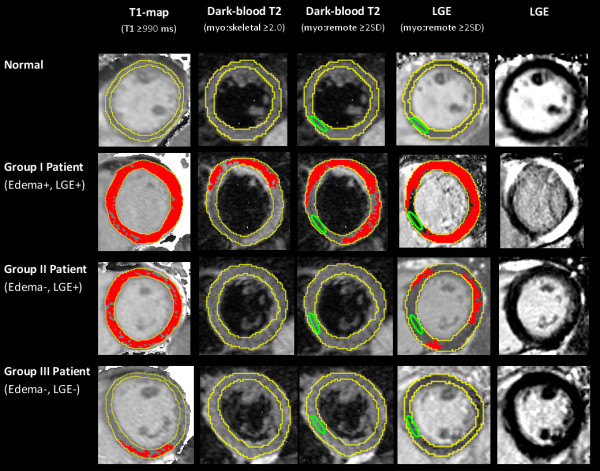
**T1-maps detected the location and extent of myocardial injury in acute myocarditis.** Areas of myocardial injury (red) as detected by computer-aided analysis of T1-maps, T2-weighted and late gadolinium enhanced (LGE) images in a normal subject (row 1) and patients with acute myocarditis (rows 2–4). Red indicates areas of myocardium with values above the stated threshold for each method. Green contour marks the position of reference region of interest (ROI). Skeletal muscle ROI for T2W images not shown. Note that a T1 cut-off of ≥ 990 ms detected larger areas of injury than corresponding T2W and LGE imaging analysis.

In order to assess whether T1-mapping offers any incremental value over conventional CMR techniques (T2W, LGE) in the detection of myocarditis, patients were further stratified into subgroups according to their diagnostic certainty based on edema (T2W) and LGE imaging as used in typical clinical settings (Figure 2). CMR findings and patient characteristics in each subgroup are presented in Tables 1 and 3.

**Table 3:** Baseline characteristics of patient subgroups compared to normal controls

	**Normal volunteers (n = 50)**	**Group I patients (Edema+, LGE+) (n = 41)**	**Group II patients (Edema-, LGE+) (n = 12)**	**Group III patients (Edema-, LGE-) (n = 7)**
Age (years)	41 ± 13	37 ± 12	46 ± 12	52 ± 13
Female, n (%)	13 (26)	68 (20)	4 (33)	3 (43)
Troponin I (μg/L)	n/a	9.60 (3.40 to 21.00)*	0.54 (0.25 to 4.19)	0.48 (0.19 to 1.29)
Coronary angiogram performed, n (%)	n/a	25 (61)	10 (83)	7 (100)*
Median (IQR) time from symptoms to CMR (days)	n/a	2 (1 to 5)	3 (2 to 7)	3 (2 to 5)

Values are mean ± SD, n (%), or median (interquartile range). *significantly different from other patient subgroups; otherwise no statistical differences in characteristics amongst subgroups.

Group I patients (edema+, LGE+) demonstrated both edema and LGE patterns typical of myocarditis. They had more severe myocardial injury, as evidenced by a significantly higher Troponin I level and lower EF than other patient subgroups and controls (all p < 0.05). Group I patients also had significantly more edema and LGE compared to controls and other patient subgroups (all p < 0.05).

Group II patients (edema- LGE+) did not fulfill criteria for edema but demonstrated typical LGE patterns. Reasons included not meeting current T2 SI cut-off values for edema despite good image quality (n = 8), and non-diagnostic image quality due to poor breath-holding (n = 2) or tachyarrhythmia (n = 2). These patients suffered less severe myocardial injury, with a significantly lower median Troponin I level, smaller areas of LGE than Group I patients (all p < 0.05) and a mean EF no different from controls.

Group III patients (edema-, LGE-) did not demonstrate edema or LGE on CMR. Three patients with difficulty breath-holding had non-diagnostic dark-blood T2W and LGE (including single shot LGE) imaging, while the rest did not demonstrate findings despite good image quality. These patients had a very low median Troponin I level (0.48 μg/L) and a mean EF no different from controls.

**Figure 3:**
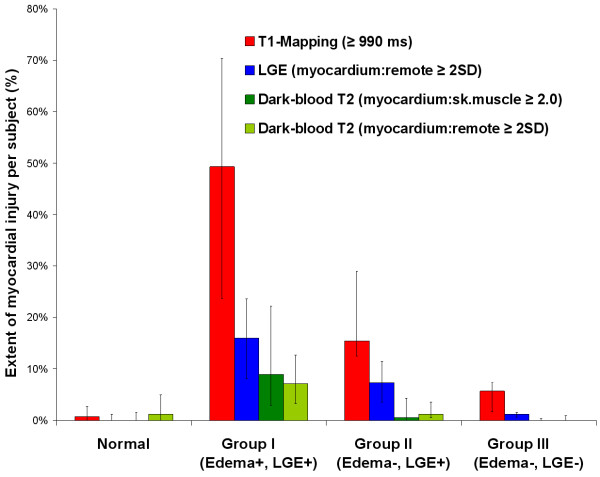
**Extent of myocardial injury detected by T1-mapping, T2W and LGE imaging in acute myocarditis.** T1-mapping detected a significantly larger extent of myocardial injury in all patient subgroups compared to controls (all p < 0.0001), and compared to T2W and LGE imaging within all patient subgroups (all p < 0.001). Bar graphs represent the median extent of injury within segments per subject; error bars mark the interquartile range.

**Figure 4:**
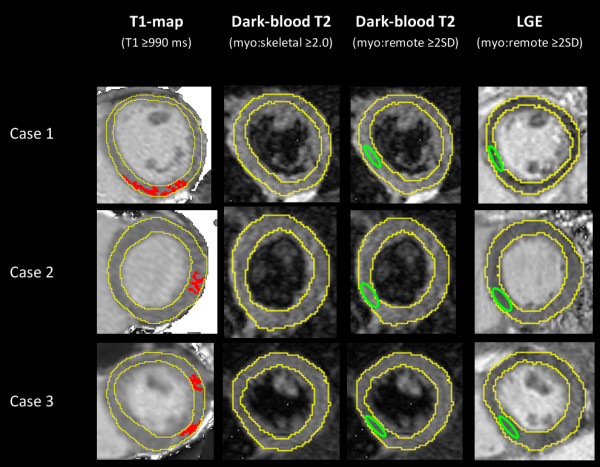
**T1-mapping located small areas of myocardial injury (red) when T2W and LGE imaging were negative.** This figure shows computer-aided analyses of CMR images from three representative cases of Group III patients (edema-, LGE-) with small rises in Troponin I. T1-mapping detected small focal areas of injury in 6 out of the 7 patients in this group, in at least 2 contiguous short-axis slices or 2 orthogonal planes that were not due to artifacts or poor T1 fit. Green contour marks the positioning of reference region of interest (ROI). Skeletal muscle ROI for T2W images not shown.

T1-mapping, using a threshold of T1 ≥ 990 ms [8], was able to detect a significantly larger extent of myocardial injury compared to T2 and LGE imaging in all patient subgroups and compared to controls (all p < 0.05; Table 1 and Figure 3). These findings demonstrate more global involvement in myocarditis than conventional methods (T2W, LGE) show. In Group II (edema-, LGE+) patients, T1-mapping was able to detect areas of acute injury in all 12 out of 12 cases, representing an additional 20% of cases when dark-blood T2-weighted imaging did not provide a diagnosis of edema, improving the PPV from the original 90% (based on the “T2 or LGE” combination representative of Group II; Table 2) to 98% (based on the “T1 and LGE” combination for the 4 patients with non-diagnostic dark-blood T2 images) and also 98% for the 8 patients with good quality dark-blood T2 images (based on the “Any 2 of 3: T1-mapping, Dark-blood T2 or LGE” combination). Further, in Group III (edema-, LGE-) patients, T1-mapping was able to detect small focal areas of injury in 6 of the 7 cases (an additional 10% of the cohort), in at least 2 contiguous slices or 2 orthogonal planes, and not due to artifacts or poor T1 fit (Figure 4). This improved the diagnostic confidence in Group III from the previous NPV of 51% for ruling out myocarditis (based on negative findings on T2 and LGE) to now a PPV of 90% for the 3 patients with suboptimal images (based on the use of T1-mapping as a single criterion) or 85% for the patients with good T2W and LGE images (now based on the “Any 1 of 3: T1-mapping, T2 or LGE” combination). Overall, T1-mapping significantly improved the diagnostic confidence in an additional 30% of cases when at least one of the conventional methods (T2W, LGE) failed to identify any areas of abnormality.

### Ability of native T1-mapping to display the patterns and distribution of myocardial injury

**Figure 5:**
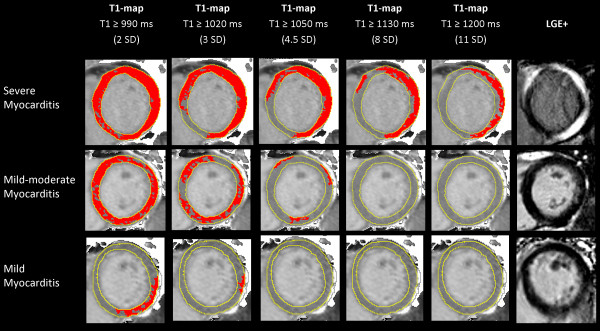
**T1-maps using incremental thresholds demonstrate the predominantly non-ischemic pattern of injury across a spectrum of acute myocarditis.** Red indicates areas of myocardium with a T1 value above the stated threshold of at least 40 mm^2^ in contiguous area. T1 threshold of 990 ms was previously validated for the detection of acute myocardial edema; other thresholds were selected for illustrative purposes.

Further information on tissue characterization may be extracted from native T1-maps using incremental thresholds (Figure 5; selected T1 thresholds shown for illustrative purposes). These revealed the underlying pattern of myocardial injury, similar to that typically seen on LGE imaging.

In cases of severe myocarditis (Figure 5, top row), T1 ≥ 990 ms revealed global myocardial injury; however, using incremental thresholds, it became evident that the areas with the highest T1 values were localized to the lateral wall in a midwall or subepicardial pattern, similar to the areas enhancing on LGE images. This is consistent with the finding that patient segments with LGE had significantly higher T1 values (1042 ± 66 ms), and patient segments without LGE (from Groups I-III) had less elevated T1 values but still >2SD above normal (993 ± 56 ms vs. 946 ± 23 ms in controls; all p < 0.0001), suggesting that areas without apparent LGE were also involved. On the other hand, in cases of low-grade myocarditis with low troponin levels (Figure 5, bottom row); while using a high T1 threshold (e.g. 1200 ms) would not have detected the changes, the threshold of T1 ≥ 990 ms allowed immediate visualization of the area of abnormality to the inferolateral wall in a predominantly subepicardial or midwall pattern.

Thus, having a range of T1 thresholds (starting at 990 ms) allows the simultaneous quantification of myocardial involvement as well as visualization of the topography of injury within the affected myocardium, providing important information such as the presence of a non-ischemic pattern of injury.

## Discussion

In this work, we have shown for the first time that, in acute myocarditis, whole-heart T1-mapping: (1) can diagnose extra cases with small areas of focal injury not detected by T2W and LGE imaging; (2) detects a larger extent of myocardial involvement than T2W and LGE methods without increasing the number of false positive findings, and; (3) can demonstrate patterns of non-ischemic injury similar to LGE imaging, without the need for exogenous contrast agents.

### Insights from T1 characterization of acute myocarditis

Consistent with the current understanding of acute myocarditis, the findings of significantly increased T2 SI and T1 times in these patients support the notion that edema and inflammation are major components in the acute pathophysiologic process [1,14]. While acute edema is known to increase native T1 relaxation times [8,15], there may be additional changes, such as the distribution of free water fractions in the intra- and extra-cellular compartments, that are more readily detected by T1-mapping [15] than T2W methods. ShMOLLI T1-mapping is more sensitive to disease (SN 90%, SP 88%) compared to T2W (SN 48%, SP 86%) and LGE (SN 72%, SP 97%) imaging using the stated thresholds, with still very good specificity [9]; this may be attributed to its tight normal range with low variability [7] and additional sensitivity to disease due to magnetization transfer effects [16], which make it an excellent imaging biomarker. The results from this study demonstrated that the higher sensitivity of T1-mapping did not result in significantly more false positive findings in normal controls (Figure 3). These and the fact that T1-mapping directly quantifies myocardial changes without the need for reference ROIs may explain why T1-mapping was able to detect larger areas of myocardial involvement than T2W and LGE imaging.

As a method for imaging edema, T1-mapping may replace dark-blood T2 with statistically better diagnostic performance in detecting changes of acute myocarditis as a single criterion (T1-mapping vs. Dark-blood T2; p < 0.05; Table 2), or in combination with LGE (T1-mapping and LGE vs. Dark-blood T2 and LGE, p = ns, with “T1-mapping and LGE” demonstrating superior or equal numerical results on all diagnostic parameters of SN, SP, Accuracy, PPV, NPV). ShMOLLI T1-mapping can be particularly useful when conventional dark-blood T2W imaging fails (either due to tachyarrhythmia, the patient’s inability to perform long breath-holds, the technique not sensitive enough, or skeletal muscle inflammation).

We present for the first time how T1-mapping permits quantification of the extent of myocardial involvement as well as visualization of non-ischemic patterns of injury in myocarditis. In many cases, a T1 threshold of ≥ 990 ms [8] demonstrated that acute myocarditis is a global process, within which there are focal areas of even higher T1 values. These areas are characterized by a predominantly subepicardial/midwall pattern or focal islands of high T1 within the myocardium, typically in the lateral or inferior wall. These observations are consistent with previous work which demonstrated that acute myocarditis starts as a focal process which then spreads globally [17,18]. Once seeded within the myocardium, it is possible that the wavefront of myocardial injury spreads by direct extension away from the initial focus (for example, in both a clockwise and counter-clockwise direction on a slice), until the entire myocardium is involved (Figure 5); this temporal pattern of injury may be confirmed in future studies by performing T1-mapping at serial time points within the first 14 days of acute myocarditis, similar to the original study using early gadolinium imaging which demonstrated this process [17]. Even in some cases of severe myocarditis, while an area may appear normal on LGE imaging (Figure 5, top row), serial T1-maps starting at T1 ≥ 990 ms revealed that the entire myocardium was involved, only that the LGE-negative area had less elevated T1 values. This is supported by the findings that, while patient segments with LGE had significantly higher T1 values, patient segments without LGE (from Groups I, II and III) still demonstrated significantly higher T1 values compared to controls (993 ± 56 vs. 946 ± 23 ms; p < 0.0001), suggesting that these areas were also likely involved but perhaps to a milder degree and presumably without significant necrosis. Further, very small foci of myocarditis may be beyond the resolution of LGE, and techniques such as T1-mapping may offer additional advantage. For instance, the PSIR sequence for LGE in this study offers a resolution of 2.0 × 1.5 × 8.0 mm, whereas the resolution of the T1-map has a voxel size of 0.9 × 0.9 × 8.0 mm based on the two-fold interpolation of the IR-weighted images. Thus, T1-maps with incremental thresholds not only can display the predominant non-ischemic pattern of myocarditis, but may also provide additional insights such as the wavefronts and truer extent of myocardial involvement.

### Clinical implications

T1-mapping with incremental thresholds revealing patterns of myocardial injury has important clinical implications, as there is now a way to extract information similar to LGE imaging without the need for exogenous contrast agents. T1-mapping can effectively act as a method for both edema and LGE pattern imaging. In the future, it may be possible to perform a gadolinium-free CMR protocol using cine and T1-mapping, with T1-mapping as a single criterion demonstrating around 90% in sensitivity, specificity, accuracy, PPV and NPV. If greater specificity and PPV were desired, an alternate approach could be to combine T1-mapping with dark-blood T2W imaging (refer to Table 2). If the patient fulfilled positive criteria for both T1-mapping and dark-blood T2-weighted imaging (taking into account that the patterns and distribution of injury are consistent with myocarditis and not, for example, an infarction), this has a PPV of 97% for acute myocarditis, so one may argue that the patient needs no administration of gadolinium for further LGE imaging, since adding LGE with a subsequent positive finding (now the “T1-mapping and Dark-blood T2 and LGE” combination criteria) has a PPV of 96%, which does not improve the previous PPV of 97%. However, if “T1-mapping and Dark-blood T2” were negative, this only has a NPV of 61% for ruling out myocarditis, hence if the clinical suspicion remains, gadolinium may be administered in attempt to either obtain a possible diagnosis based on a third criterion (now according to the “Any 1 of 3” combination in Table 2) or to further rule out disease. In this case, if LGE turns out to be positive in a pattern compatible with myocarditis, then the PPV is 85%. Such an approach should be tested against other cardiac pathologies in a wide spectrum of clinical presentation to further investigate the ability of T1-mapping to distinguish diseases in combination with other CMR techniques. It does hold promise for abbreviating CMR protocols and may be particularly useful for patients in whom gadolinium contrast is contraindicated.

For T1-mapping to successfully translate into clinical practice, its ability to provide information on the entire left ventricle in all territories would be important, especially for a disease such as myocarditis in which pathology may be patchy and global. Immediate visual color T1-maps and those based on validated thresholds to detect disease with minimal extra post-processing steps [8,19,20] may facilitate the adoption of T1-mapping into clinical practice.

### Limitations

In line with local and international scientific statements and guidelines, endomyocardial biopsy is not routine clinical practice in both hospitals, as it is of low diagnostic yield and sensitivity for most cases of myocarditis with inherent procedural risks [21-27]; as such, direct histopathologic correlation to the imaging findings is not available. As most of the patients (93%) had a preceding viral or inflammatory illness, after exclusion of obstructive coronary disease and alternate diagnoses (e.g. Takotsubo, HCM, DCM, MI) together with their CMR findings, acute myocarditis is the most likely diagnosis for the majority of the cohort. The study protocol did not include early gadolinium enhancement (EGE) for practical reasons, as our protocol was already lengthy due to whole-heart tissue characterization for multiple sequences. Thus, we did not test the ability of T1-mapping to replace EGE imaging. The original aim of the study was to validate the novel T1-mapping technique in a selected cohort with suspected myocarditis defined by clinical features, so none of the Lake Louise (LL) criteria was used as inclusion criteria. It has also recently been shown that the sensitivity of “T2 and/or LGE” was significantly higher than the LL criteria (91% vs 80%), and omission of EGE did not alter the overall diagnostic accuracy [28]. For Group III (edema-, LGE-) patients, while T1-mapping did detect small areas of injury, it is difficult to ascertain the exact etiology of those findings, especially in patients without a viral prodrome; causes may include a ruptured coronary plaque or other causes of myocardial injury. Increased native T1 values may be seen in a variety of cardiac conditions [29-36] and it would be important for future studies to test the ability of native T1-mapping to distinguish different pathologies systematically in larger clinical studies. T1-thresholds (such as T1 ≥ 990 ms [8]) for distinguishing disease from normal may be platform-specific [37]. Further, the incremental T1 thresholds presented in Figure 5 were selected for illustrative purposes and not directly validated against severity of tissue injury, although these areas corresponded to areas of LGE; whether the degree of T1 elevation directly corresponds to the severity of injury on the histopathologic level will have to be explored in future studies. MOLLI-based T1-mapping techniques exhibit some regional variation of T1 values across the myocardium [11,38]; for the detection of subtle, localized changes, comparison to regional T1 norms may further improve the ability of disease detection.

## Conclusions

Native T1-mapping can display the typical non-ischemic patterns in acute myocarditis, similar to LGE imaging without the need for contrast agents. T1-mapping also detected additional areas of myocardial involvement and identified extra cases beyond T2W and LGE imaging, improving the diagnostic confidence when conventional methods failed to identify abnormalities. In the future, it may be possible to perform gadolinium-free CMR using cine and T1-mapping for tissue characterization and may be particularly useful for patients in whom gadolinium contrast is contraindicated.

## Abbreviations

ANOVA: Analysis of variance; AUC: Area under the curve; CAD: Coronary artery disease; CMR: Cardiovascular magnetic resonance; EGE: Early gadolinium enhancement; LGE: Late gadolinium enhancement; LVEF: Left ventricular ejection fraction; IQR: Interquartile range; MOLLI: Modified Look-Locker Inversion Recovery; PSIR: Phase-sensitive inversion recovery; ROC: Receiver operating characteristic; ROI: Regional of interest; SD: Standard deviation; ShMOLLI: Shortened Modified Look-Locker Inversion Recovery; SI: Signal intensity; SN: Sensitivity; SP: Specificity; STIR: Short-tau inversion recovery; T2W: T2-weighted; TD: Trigger delay; TE: Echo time; TI: Inversion time; TR: Repetition time.

## Competing interests

Financial competing interests.

MGF is board member, advisor and shareholder of Circle Cardiovascular Imaging Inc.

Non-financial competing interests.

US patent pending 61/387,591: SKP, MDR. SYSTEMS AND METHODS FOR SHORTENED LOOK LOCKER INVERSION RECOVERY (Sh-MOLLI) CARDIAC GATED MAPPING OF T1. September 29, 2010. All rights sold exclusively to Siemens Medical Solutions.

US patent pending 61/689,067: SKP, MDR. COLOR MAP DESIGN METHOD FOR IMMEDIATE ASSESSMENT OF THE DEVIATION FROM ESTABLISHED NORMAL POPULATION STATISTICS AND ITS APPLICATION TO CARDIOVASCULAR T1 MAPPING IMAGES.

## Authors’ contributions

VMF: contributed substantially to the conception and design of the study, recruitment of subjects, data acquisition, data processing, analysis, interpretation and drafting the manuscript; SKP contributed substantially to writing the dedicated software for data post-processing, data analyses, interpretation and generation of T1- and colored T1-maps; EDA, JMF, NN and CH contributed to data acquisition; AK contributed to recruitment of subjects; TDK, RPC, MGF, MDR have critically revised the manuscript; SN participated in the design and coordination of the study and critically revised the manuscript. All authors contributed to critical revision of, reading and approving the final manuscript.

## Supplementary Material

Additional file 1 CMR Image acquisition parameters. Click here for file
