# Supplementary material for: Native T1-mapping detects the location, extent and patterns of acute myocarditis without the need for gadolinium contrast agents
Source: J Cardiovasc Magn Reson. 2014 May 23;16(1):36. doi: 10.1186/1532-429X-16-36 (PMC4041901; doi:10.1186/1532-429X-16-36)
Supplement: Additional file 1 — CMR Image acquisition parameters. [file 1532-429X-16-36-S1.docx]

**ADDITIONAL FILE 1**

**CMR Image acquisition parameters**

Typical imaging parameters for SSFP cine imaging were: voxel size 2.0x2.0x8.0 mm, TR/TE 39.6/1.12 ms, flip angle 55^o^, matrix 192x192; ShMOLLI T1-maps are based on 5-7 images with specific TI~100-5000 ms, collected using SSFP readouts in a single breath-hold, typically: TR/TE~201.32/1.07 ms, flip angle=35º, matrix=192x144, 107 phase encoding steps, interpolated voxel size=0.9x0.9x8 mm, cardiac delay time TD=500 ms; 206 ms acquisition time for single image [1, 2]; STIR: voxel size 1.9x1.5x10.0 mm, matrix=256x166, effective echo time TE=61ms, effective repetition time TR=2 RR intervals during breath-hold, flip angle 180^o^, echo spacing 6.74 ms, TI=170 ms, dark blood thickness 200%, dark blood flip angle 180^o^, turbo factor 25, echo trains per slice=7 [3, 4]; phase-sensitive inversion recovery sequence: voxel size 2.0 x 1.5 x 8.0 mm, matrix 144x256, TR/TE=800.20/3.36ms, flip angle 25^o^) [5] .

SSFP = Steady-state Free Precession; ShMOLLI = shortened modified Look-Locker inversion recovery; STIR = short-tau inversion recovery; TD = trigger delay; TE = echo time; TI = inversion time; TR = repetition time

**Table 4:** Diagnostic Performance of CMR Tissue Characterization Methods in the Detection of Suspected Acute Myocarditis (without rejecting image artifacts)

| **Tissue Criteria** | **Sensitivity (%)** | **Specificity (%)** | **Accuracy (%)** | **PPV (%)** | **NPV (%)** |
| --- | --- | --- | --- | --- | --- |
| **Individual** |  |  |  |  |  |
| T1-mapping | 92 | 76 | 85 | 82 | 88 |
| Dark-blood T2 | 48 | 86 | 66 | 81 | 58 |
| LGE^§^ | 72 | 97 | 81 | 98 | 67 |
| **Combination (with LGE)** |  |  |  |  |  |
| Dark-blood T2 and LGE (2 out of 2)†‡ | 45 | 97 | 64 | 96 | 51 |
| Dark-blood T2 or LGE (Any 1 of 2) | 75 | 86 | 79 | 90 | 67 |
| T1-mapping and LGE (2 out of 2)† | 70 | 100 | 81 | 100 | 66 |
| T1-mapping or LGE (Any 1 of 2) | 93 | 69 | 84 | 84 | 86 |
| T1-mapping, Dark-blood T2 or LGE) (Any 1 of 3) | 93 | 63 | 82 | 81 | 85 |
| T1-mapping, Dark-blood T2 or LGE (Any 2 of 3) | 73 | 91 | 80 | 94 | 67 |
| T1-mapping and Dark-blood T2 and LGE (3 out of 3) | 45 | 100 | 65 | 100 | 52 |
| **Combination (without LGE)** |  |  |  |  |  |
| T1-mapping and Dark-blood T2 (2 out of 2)‡ | 48 | 94 | 69 | 91 | 60 |
| T1-mapping or Dark-blood T2 (Any 1 of 2) | 92 | 68 | 81 | 78 | 87 |
| T1-mapping: myocardial injury is detected when T1 is ≥ 990 ms; Dark-blood T2-weighted imaging: edema is diagnosed when the T2 SI ratio (T2 SI _myocardium : skeletal muscle_) is ≥ 2:1; Late gadolinium enhancement (LGE) is detected when myocardial SI is ≥ 2 SD above mean SI of remote myocardium.  For each technique, only contiguous areas of myocardium ≥40 mm^2^ above the stated threshold were considered relevant; involvement of ≥5% of any segment on a per-subject basis was the threshold used for comparison of methods. PPV = positive predictive value; NPV = negative predictive value | | | | | |

**References**

1. Piechnik SK, Ferreira VM, Dall'Armellina E, Cochlin LE, Greiser A, Neubauer S, Robson MD: Shortened Modified Look-Locker Inversion recovery (ShMOLLI) for clinical myocardial T1-mapping at 1.5 and 3 T within a 9 heartbeat breathhold. *J Cardiovasc Magn Reson* 2010, 12:69.

2. Piechnik S, Ferreira V, Lewandowski A, Ntusi N, Banerjee R, Holloway C, Hofman M, Sado D, Maestrini V, White S, et al: Normal variation of magnetic resonance T1 relaxation times in the human population at 1.5T using ShMOLLI. *J Cardiovasc Magn Reson* 2013, 15:13.

3. Friedrich MG, Sechtem U, Schulz-Menger J, Holmvang G, Alakija P, Cooper LT, White JA, Abdel-Aty H, Gutberlet M, Prasad S, et al: Cardiovascular Magnetic Resonance in Myocarditis: A JACC White Paper. *J Am Coll Cardiol* 2009, 53:1475-1487.

4. Simonetti OP, Finn JP, White RD, Laub G, Henry DA: "Black blood" T2-weighted inversion-recovery MR imaging of the heart. *Radiology* 1996, 199:49-57.

5. Kellman P, Arai AE, McVeigh ER, Aletras AH: Phase-sensitive inversion recovery for detecting myocardial infarction using gadolinium-delayed hyperenhancement. *Magn Reson Med* 2002, 47:372-383.
